# Determinants of mutation susceptibility along the genome are largely invariant across human tissues

**DOI:** 10.1186/s13059-026-04245-1

**Published:** 2026-08-22

**Authors:** Corinna Lewis Schmalohr, Yashna Paul, Nina Bundschuh, Andreas Beyer

**Affiliations:** 1 Cologne Excellence Cluster for Aging and Aging-Associated Diseases (CECAD), University of Cologne, 50931 Cologne, Germany; 2 Institute for Genetics, Faculty of Mathematics and Natural Sciences, University of Cologne, 50923 Cologne, Germany; 3 Center for Molecular Medicine Cologne, Faculty of Medicine and University Hospital Cologne, University of Cologne, 50931 Cologne, Germany

**Keywords:** Somatic mutations, SNVs, Chromatin context, Machine learning, Mutation susceptibility, Sequence context

## Abstract

**Background:**

The propensity for accumulating somatic mutations varies along the genome, which critically influences somatic mosaicism, tumor evolution and the potential role of somatic mutations in the context of age-associated diseases. Genomic factors contributing to the variability of mutation rates have been established, including, for example, distance from the replication origin, chromatin structure and sequence context. However, their relative importance for explaining variable mutation rates along the genome as well as variable mutation rates between tissues remains elusive.

**Results:**

Here, we present a modelling strategy that integrates 146 genomic features at different scales to predict susceptibilities for point mutations in 25 human tissues along the genome. These models faithfully predict mutation rates in coding and non-coding parts of the human genome in cancer and healthy tissues, including even unseen tissue types that were not used during the model training. Our work revealed that the dependency of mutation rates on chromatin structure and other genomic features is remarkably invariant across tissues, pointing to fundamental, conserved processes underlying mutagenic processes. Local variability in mutation rates on the scale of a few base pairs is almost exclusively driven by the sequence context, whereas large-scale variability is dominated by chromatin features, gene expression and GC content.

**Conclusions:**

Our modelling strategy quantifies the relative contribution of genomic factors to mutation susceptibility, predicting mutational biases at any genomic resolution across human tissues, and provides a basis for better understanding tumor evolution and age-related diseases.

**Supplementary Information:**

The online version contains supplementary material available at 10.1186/s13059-026-04245-1.

## Background

Somatic mutation rates greatly vary between tissues and between different positions of the genome. This variability is determined by a combination of factors, including the mutagen/mutagenic process, chromatin structure, DNA repair efficiency, and selective pressure [1–5]. Determining the extent of this regional variation on mutability is crucial for understanding the mechanisms that maintain genome integrity and for distinguishing neutral mutations from those contributing to tumorigenesis. In addition, somatic mutations are suspected to contribute to aging, age-associated diseases and epigenetic alterations on larger genomic scales [6]. Hence, a more quantitative understanding of the processes driving somatic mutations will also aid the understanding of age-related genomic changes.

Previous studies have investigated the impact of genomic features on mutation susceptibility at different genomic scales. For example, at a large genomic scale, models incorporating different subsets of replication timing, GC content, DNA repair, and histone marks have consistently identified these features as major determinants of regional mutation rates [7–12]. More recent studies have refined these approaches to single-base resolution, demonstrating that sequence context, replication timing, repetitive DNA structures, and gene expression levels have individually been identified as major predictors of mutation rate variation [13, 14]. Other methods either estimate germline mutation rates based on sequence context without incorporating chromatin or epigenomic features [15], operate at megabase resolution to predict regional mutation burden [16], or test for enrichment of mutations over predefined annotation windows rather than mutation rate prediction [17].

However, while the sensitivity of technical assays for the detection of somatic mutations is rapidly increasing [18], fundamental understanding regarding the mechanisms determining mutational biases along the genome is still lacking. That is, even though many genomic and epigenomic features contributing to mutation susceptibility have been identified, their relative and potentially confounding effects remain unclear [5, 19]. Furthermore, the relative contribution of sequence context-based mutational signatures compared to other genomic features to the total variation in mutation rate along the genome has not been conclusively studied.

To address these gaps, we developed tissue-specific, single-base pair resolution machine learning models to predict mutation susceptibility across the coding and non-coding parts of the human genome. Here, mutational susceptibility is defined as *the relative probability that a given genomic position is observed to harbor a somatic mutation in bulk sequencing data.* Thus, the goal was to predict regional biases in the genome that increase or decrease the probability of acquiring a somatic mutation. Importantly, the set of total mutations in any given tissue (or cell) depends on this mutational susceptibility as well as the mutational burden (i.e. the total number of mutations in a sample). This also means that, in a given sample, mutations may still, with a low probability, occur in regions with low theoretical mutation susceptibility. Our approach integrates a comprehensive set of up to 146 tissue-specific (e.g., DNA accessibility) and tissue-independent (e.g., GC content) genomic and epigenomic features to assess their relative contribution to somatic mutability. The application of these models to healthy tissues and tumor specimens revealed that the relationships between genomic features and mutation susceptibility are remarkably stable across tissues. Further, we quantified the importance of sequence context compared to other genomic features for predicting mutation rates at any scale, revealing that sequence context alone is sufficient for predicting small scale (< 100 bp) variation in mutation susceptibility, while other genomic features dominate at greater scales (> 1kpb). Based on these findings we developed an integrated, tissue-agnostic model for predicting mutation rates along the entire human genome at any genomic resolution.

## Results

### Training and evaluation of mutation model based on genomic features

**Fig. 1:**
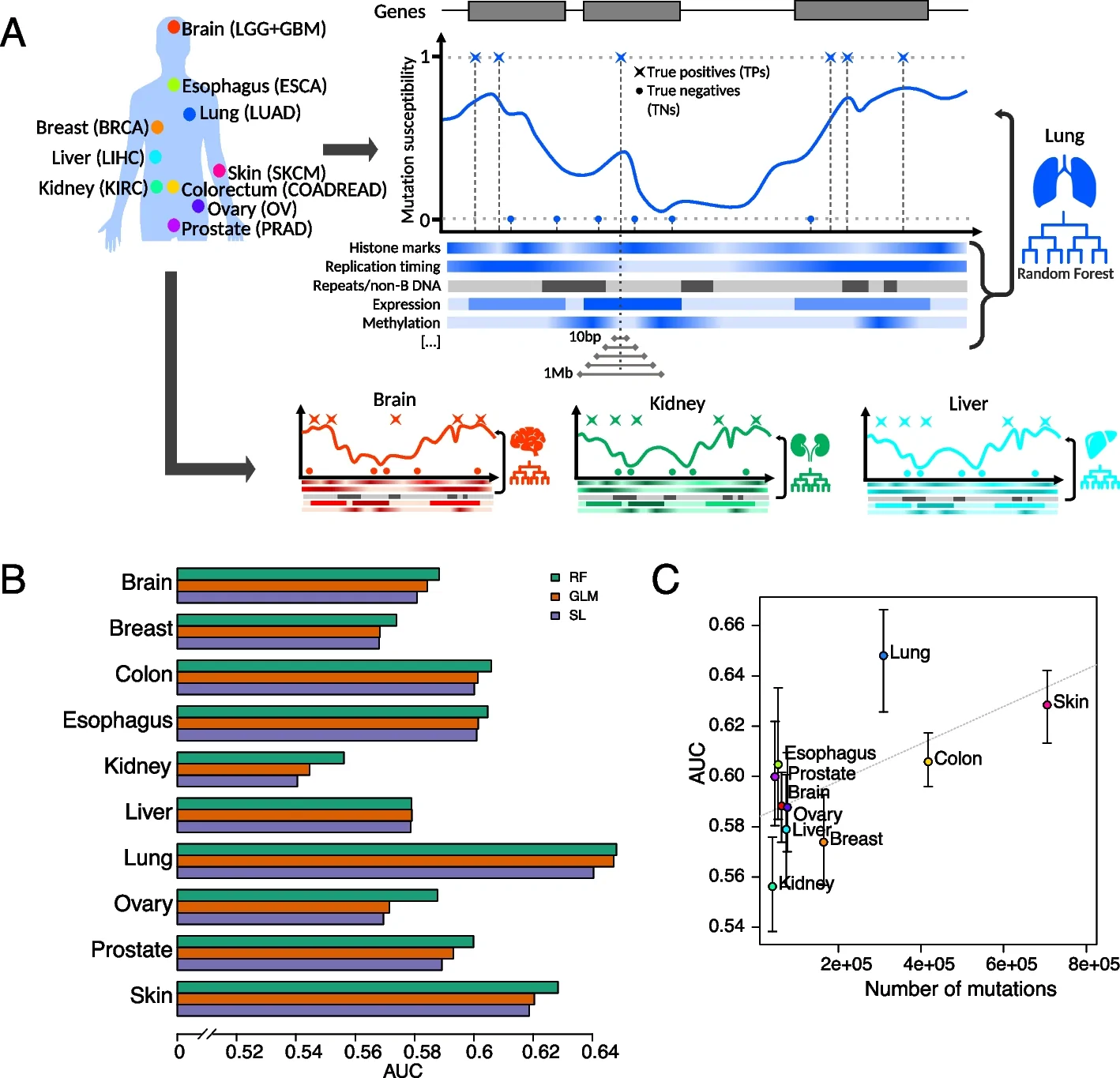
Creating a model predicting somatic mutation susceptibility along the exome. **A** Schematic representation of the modeling approach: Genomic features were used to differentiate mutated (TP) from non-mutated (TN) positions using random forest (RF) based on tissue-specific predictors where applicable. The RF model was then used to predict single-basepair mutation susceptibility along the exome. This process was repeated for each of the ten tissues. Tissue-specificity of mutations, predictors and models are represented by tissue colors. Non-tissue specific predictors are depicted as grey. **B** Model performance (area under the ROC curve, AUC) for random forest (RF), generalized linear model (GLM) and stability selection (SL). **C** RF model performance with relation to training data size. Error bars represent the standard deviation of the AUC estimates across CWCV folds while the dots represent the estimate based on concatenated predictions

To systematically investigate the determinants of somatic mutation susceptibility across the coding exome and genome, we trained models to predict mutational propensity at base-pair resolution, based on chromatin context and local sequence (Fig. 1A). Models were trained using somatic mutation data for ten cancer tissues—brain, breast, colon, esophagus, kidney, liver, lung, ovary, prostate, and skin—from the Pan-Cancer Atlas [20]. Overall, 146 genomic and epigenomic features (evaluated across multiple genomic window sizes, i.e. 1 bp, 10 bp, 100 bp, 1 kb, 10 kb, 100 kb, and 1 Mbp) were considered per model. These features include factors representing genome organization (replication timing, Hi-C data, GC content, DNA-accessibility, distance to centromeres and telomeres), transcriptional activity (expression, histone marks, methylation, transcription factor binding), as well as sequence features (conservation, non-B DNA) (Additional file 1: Table S1). Among these, 22 were tissue-specific (e.g. gene expression, histone modifications, chromatin accessibility), allowing us to account for tissue-dependent mutational influences. Initially, we trained models specifically for the coding exome (referred to as “exome” in the following), later for the entire human genome (Methods). To minimize the influence of positive selection in the training data, we excluded positions that were mutated more than once (i.e., in more than one patient) from the training data (Additional file 2: Fig. S1). This resulted in 1,170,928 mutated positions (positive training examples, TP) across all tissues, with per-tissue mutation counts ranging from 22,430 (kidney) to 456,455 (skin) (Additional file 2: Fig. S2). Matching negative training examples (TN) were sampled from the non-mutated exome such that the number of sequence context pentamers were equal between positive and negative examples. The impact of sequence context on mutation rate was explicitly modeled in a subsequent approach.

We reasoned that in the exome-based model, the impact of some genomic features may be underestimated, since they play only a minor role within the coding region. For instance, non-B DNA structures can interfere with regular transcriptional activity and are therefore subject to negative selection in exons. Similarly, most TFBS are located outside of coding regions. To account for this, we defined some predictors (e.g. GC content, histone modifications, CTCF binding sites, ATAC-seq peaks etc.) over genomic regions of varying size (with window sizes increasing in powers of ten, e.g., 1 bp, 10 bp, …, 1 Mb), assessing their effects on mutation susceptibilities over smaller windows as well as larger ones, even when such annotations were rare within the exome.

For the prediction of somatic mutation susceptibility based on chromatin features across the exome, we evaluated three machine learning approaches—Random Forest (RF), logistic regression (generalized linear model, GLM), and LASSO with stability selection (SL). These methods covered a spectrum of statistical and machine learning techniques suitable for high-dimensional data and feature selection.

We assessed model performance using chromosome-wise cross-validation (CWCV), a 22-fold cross-validation scheme in which each of the 22 autosomes served as the test set in turn, to minimize spatial autocorrelation bias. RF models outperformed GLM and SL in most tissues, with statistically significant improvements (DeLong's test, two-sided, Benjamini-Hochberg, BH, adjusted *p* < 0.05) over both alternatives in 6 out of 10 tissues (breast, colon, kidney, ovary, prostate, skin); RF significantly outperformed SL in brain and lung but was statistically indistinguishable from GLM in those two tissues, while in liver and esophagus the three approaches did not differ significantly (Fig. 1B). All three approaches significantly outperformed random prediction across all tissues, achieving areas under the ROC curve (AUC) between 0.54 and 0.7 (one-sample t-test, BH-adjusted *p* < 1 × 10⁻^1^⁰; Additional file 2: Fig. S3A-C). Variation in model performance across tissues exceeded variation across methods, suggesting that tissue-specific mutation spectra contribute more strongly to the AUC than modeling strategy. The performance advantage of RF where present likely reflects its capacity to capture non-linear effects and interactions among predictors. Further, we noted that the performance of the RF model on the training and test data was very similar, strongly suggesting that RF was not overfitting (Additional file 2: Fig. S3D). Importantly, RF performance and feature importances were stable across CWCV folds and independent of chromosome size or other chromosome-specific features (Additional file 2: Figs. S4, 5). Therefore, we decided to use the RF models for all further analyses.

### Model performance is not affected by sample size

We observed clear differences in model performance between tissues (Additional file 2: Fig. S6A, B). Tissues with more mutations in the training data (i.e., colon, lung, and skin) achieved better performances than other tissues (Fig. 1C). We suspected that the difference in performance between tissues could arise from either a variable number of mutations per patient (Additional file 2: Fig. S7A), or a variable number of included patients per tissue (Additional file 2: Fig. S7B), both resulting in varying data availability between tissues.

To test whether the differences in model performance between tissues was due to data size, we conducted two types of downsampling analyses: first, we randomly downsampled *mutations* for each tissue to match the tissue with the fewest mutations (i.e., kidney with 22,430 mutations); second, we randomly downsampled *samples* (i.e., patients) to match the tissue with the lowest number of samples (i.e., ovary with 180 samples). Note that in the second approach, the total number of mutations may differ between tissues, since the samples have varying numbers of mutations. Both approaches were supposed to simulate a scenario in which true mutations remained undetected due to undersampling. We then re-trained models and computed AUCs based on CWCV as before. Downsampling had no marked effect on model performance (Additional file 2: Fig. S7C). Thus, the fact that mutations were more predictable in some tissues than in others was not explained by dataset size.

### Hypermutator samples do not strongly bias the predictions

Next, we tested if individual outlier samples with very high numbers of mutations (i.e., hypermutators) might have skewed the mutation spectrum, biasing the model performance. Hypermutator samples may include somatic clones that have exceptionally large numbers of mutations, for example due to defects in DNA polymerases or mismatch repair deficiencies, or external factors such as chemotherapy [1].

In fact, the Pan-Cancer Atlas did contain such hypermutator samples, which sometimes contributed large proportions of mutations (Additional file 2: Fig. S8A). In the brain and prostate, hypermutator samples contributed to about a third (39.1% and 29.2% respectively) of all mutations reported for those tissues. We defined samples that contributed more than 5% of the mutations for a given tissue as potentially problematic hypermutators (Additional file 1: Table S2). Mutation type distribution per tissue before and after removing hypermutator samples is shown in Additional file 2: Fig. S9A-G. While most mutation type distributions were unaffected (Additional file 2: Fig. S9B, D, F-G), some of these samples had a considerable effect on the observed mutation type spectrum, such that removing them changed the mutation type distribution (Additional file 2: Fig. S9A, C, E). To test whether these hypermutator samples might have biased the testing performance, we computed the prediction performance of the model after removing each of the top five hypermutator samples in each tissue (Additional file 2: Fig. S8B). We found that there was only one sample from the brain (“Patient 1” from the brain, TCGA-DU-6392) which noticeably influenced model performance when removed from the test data (AUC increased to 0.58 from 0.62 of the whole data). However, re-training a model without this sample did not influence model performance (Additional file 2: Fig. S8C), which underlines the robustness of the model training. Thus, including this sample only led to an underestimation of model performance in the brain, and we therefore decided against excluding it from further analyses.

### Models are predictive across tissues

**Fig. 2:**
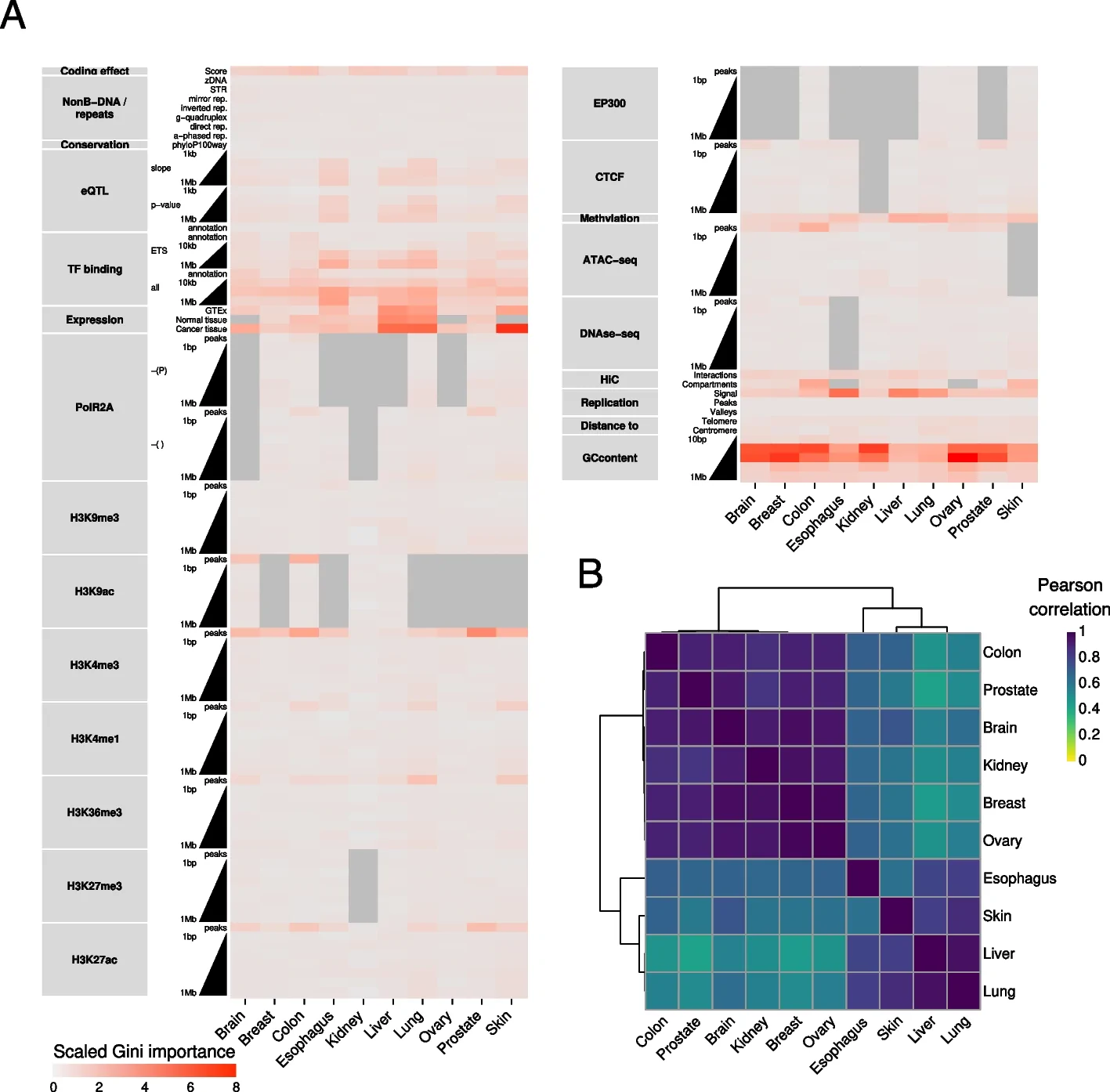
Random Forest feature importance across tissues. **A** Heatmap of Gini importances values, scaled across tissues. Each row represents one genomic feature that acted as a predictor in our models. Grey fields represent predictors whose measurements were not available for a tissue. **B** Pearson correlation of predictor importance (scaled Gini importance; Panel A) across the 10 tissues shows that the same genomic features tend to be predictive of mutation rate across tissues

Next, we used the RF gini importance to determine which genomic features were most influential in predicting the mutation susceptibility along the genome. As shown previously, we also observed that the genomic features such as GC content, replication timing (replication wave signal), DNA methylation, H3K27ac, H3K4me3, H3K9ac, gene expression, and transcription factor binding site (TFBS) density were top-ranking features in most tissues (Fig. 2A) [7–9, 13]. Feature importance scores were correlated well between tissues (Fig. 2B), suggesting that the same genomic features tend to be predictive across tissues.

**Fig. 3:**
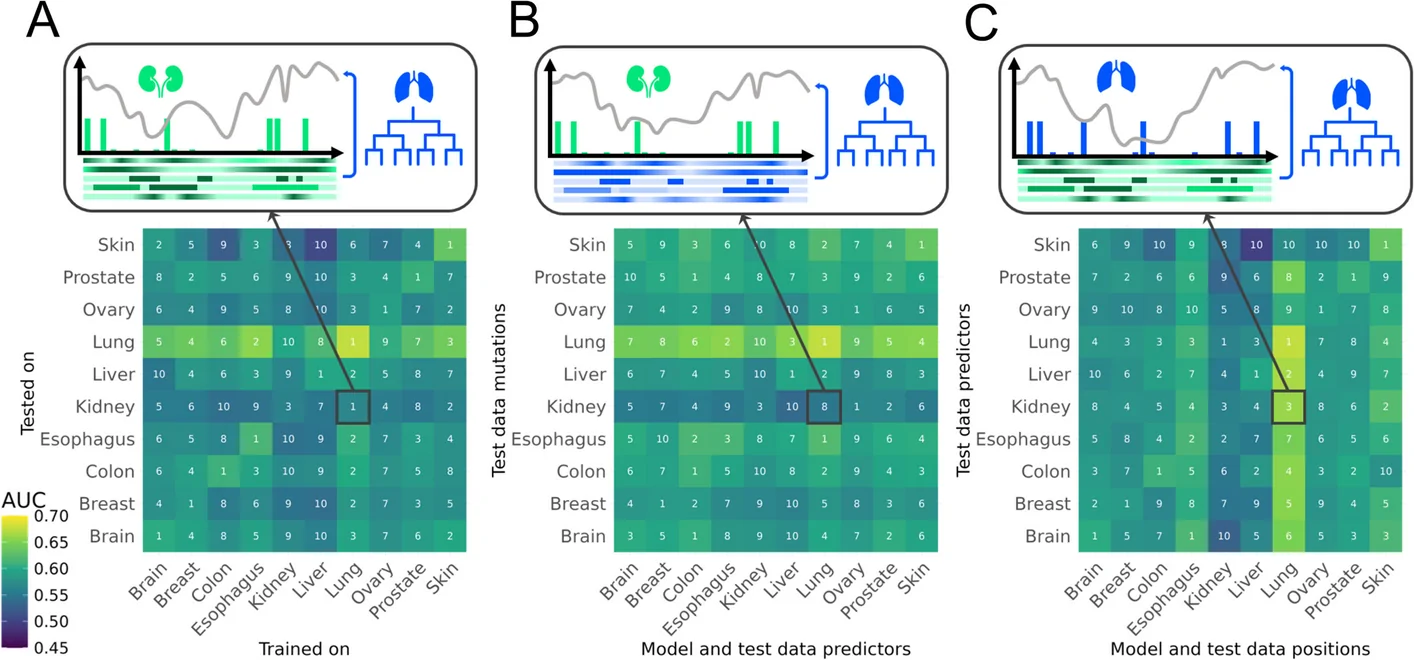
Cross-tissue application of the Random Forest models. **A** Model performance (AUC based on CWCV, fill color) of RF models trained per tissue (x-axis) to the data of all other tissues (y-axis). The schematic above the heatmap visualizes the approach for one example field, where the lung model was applied to kidney data. For each tissue, we row-wise ranked the AUC values of models trained on predictors from the 10 tissues, assigning ranks from 1 to 10, with lower ranks indicating better performance (numbers in the heatmap fields). **B** Each tissue-specific model (x-axis) was applied to predict mutations in the tissue indicated on the y-axis, but using predictors originating from the same tissue that the model was trained on (x-axis). For example, (see schematic) the lung model was applied to differentiate kidney TPs and TNs, but using lung predictors. The model performance using the ‘wrong’ features is not substantially worse compared to using the correct features (compare Panel A). The horizontal color bands indicate that the model performance is mostly dependent on the tissue in which the mutations occur. **C** Analogous to (B), models from each tissue were applied to positions from the same tissue (x-axis), but using predictors from another tissue (y-axis). Contrary to A and B, the numbers in the heatmap fields indicate the column-wise rank of the AUC value. For example (see schematic), the lung model was used to differentiate lung TPs and TNs, but using predictors from the kidney. The vertical color bands further support the notion that the source of the predictors does not majorly determine model performance

The marked cross-tissue correlation of feature importances suggested that the models themselves could be similar between different tissues. To test that notion, we applied models trained on one tissue to predict mutation susceptibilities in another tissue. This cross-tissue application revealed that the model from one tissue could be applied to the data from a different tissue (test data) with only a minor loss of accuracy (based on CWCV; Fig. 3A). Intriguingly, the prediction performance was much more dependent on the target tissue (i.e., the tissue in which mutations were predicted) than the tissue in which the models were trained, as indicated by horizontal bands in Fig. 3A. For instance, a model trained using predictors from breast tissue performed better on lung data (AUC = 0.63) than on the breast tissue data itself (AUC = 0.57). This differs from the expectation that models perform best on their training data, and are less accurate on an independent dataset. This effect was particularly pronounced for mutations in the lung, which could be relatively well predicted by any other tissue-specific model. This pattern persisted even when repeating the cross-tissue analysis on data downsampled to the tissue with the fewest mutations (Additional file 2: Fig. S10B), ruling out differences in data size between tissues as an explanation for this observation. Thus, whether or not a mutation was predictable depended on the tissue in which it occurred, while the models trained on the different tissues were remarkably similar.

Next, we tested whether the tissue-to-tissue variation in prediction performance could be explained by variable data quality of the predictors using two permutation schemes: (i) using the features from the tissue in which the models were trained to predict mutations in a different tissue, e.g. the model trained on brain was applied to predict mutations in the breast, using features from the brain (Fig. 3B); and (ii) using the tissue-matching model for predictions, but features from a ‘wrong’ tissue, e.g. using the model trained on breast data to predict mutations in the breast, but using features from the brain (Fig. 3C). In both cases the model performance did not decline substantially when replacing predictors, but was rather associated with the target tissue, suggesting that the mutations themselves, and not the predictors, determined the varying tissue performance.

### Mutation type determines predictability

**Fig. 4:**
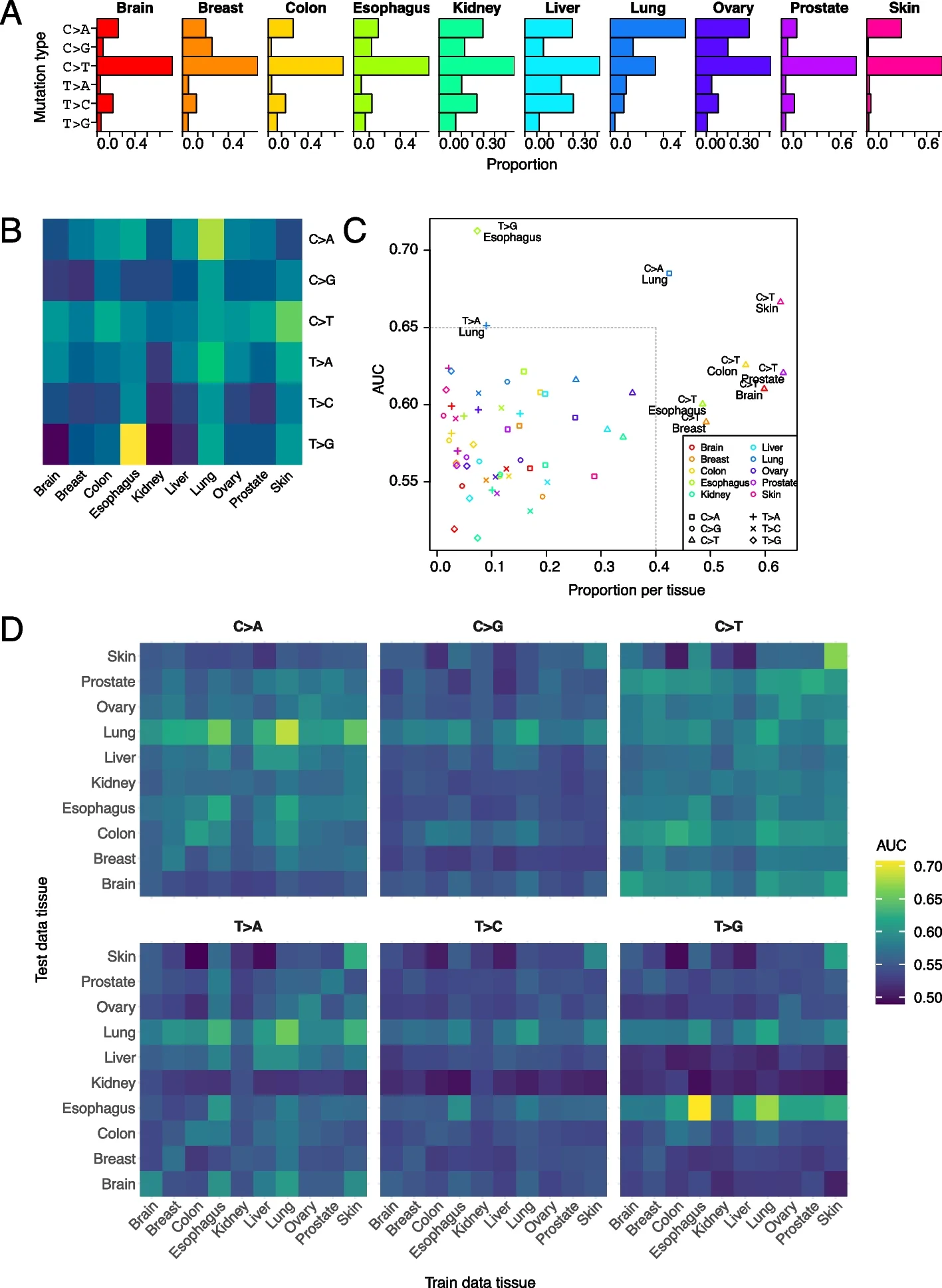
Effect of mutation type on model performance. **A** Relative frequency of substitution types present in each tissue. **B** Each tissue-specific model (trained on entire data) was tested on the subset of positions corresponding to the indicated substitution type (AUC, fill color). **C** Relationship between the prediction performance shown in (B) with the proportion of mutations that the substitution type makes up in each tissue shown in (A). Points outside the lower left quadrant (grey dashed line) were considered tissue-substitution type pairs that might be related to exceptional predictability of some tissues. **D** Analogously to the cross-tissue analysis in Fig. 3A, we applied RF models trained on one tissue (x-axis) to the data of all other tissues (y-axis), respectively. Model performance (AUC, fill color, based on CWCV) was stratified by substitution type

The fact that we could swap models between tissues without substantially losing predictive power indicated that the overall relationship between genomic features and mutation susceptibilities across the genome is preserved in all tissues. We hypothesized that the mutation type might determine how well it can be predicted and that tissues with better model performance contain more of such easily predictable mutations. For example, mutations in the lung were particularly well predictable, even when using models trained on a different tissue (Fig. 3A). Thus, we speculated that mutations in the lung might be enriched for easily predictable mutation types. To test this hypothesis, we calculated the relative frequency of substitution types present in each tissue (Fig. 4A), and evaluated prediction performance separately for each of the six possible base substitution types, i.e., C > (A/G/T) and T > (A/G/T), by subsetting the test data of each tissue to a specific substitution type. Reverse complementary mutation types were merged respectively, because the strand on which the mutation occurred cannot be distinguished from DNA sequencing data (e.g., C > A is treated identical to G > T). This revealed that certain substitution types, for example, C > A substitutions in the lung, C > T mutations in skin, and T > G mutations could be better predicted by tissue-specific models than others (Fig. 4B). The former two were also relatively frequent mutation types and predicted with high accuracy in their respective tissues (Fig. 4A, C), suggesting that they could be responsible for the tissue-specific prediction performance observed in the cross-tissue model application (Fig. 3A). To test this, we repeated the cross-tissue analysis from Fig. 3A, but stratified the results by the six base substitution types (Fig. 4D). The resulting heatmaps expose ‘patterns of predictability’, i.e., they show which mutation types are generally better predictable across tissues and/or models as opposed to being only predictable by specific models in specific tissues. This analysis revealed remarkable relationships between mutation type and model performance, detailed in the following.

Some substitution types which were enriched in a particular tissue could be comparably well predicted by models trained on any tissue. For example, C > A substitutions in the lung could be well predicted by most models (Fig. 4D). Tobacco smoke preferentially causes C > A substitutions in the lung [1]. Hence, mutations due to tobacco smoke could be more strongly influenced by genomic features compared to spontaneous clock-like mutations that are predominant in other tissues. The fact that the horizontal band for Lung was less pronounced when stratifying for other mutation types (Fig. 4D) implies that the model performance in the lung was primarily driven by C > A substitutions. Thus, although C > A substitutions are more frequent in the lung compared to other tissues, they are caused by mechanisms that exist in several tissues and hence, their relationship to genomic features is learnable across tissues. Similarly, C > T substitutions in the skin were well predictable by the skin-specific model (Fig. 4B) and they majorly contributed to the overall model performance in the skin (Fig. 4C). However, models trained on other tissues performed notably worse for predicting C > T substitutions in the skin, although they were capable of predicting C > T substitutions in tissues other than skin (Fig. 4D). In the skin, C > T mutations are mostly caused by UV-induced DNA damage, while C > T mutations arising from spontaneous deamination of methylated cytosines (COSMIC signature SBS1) is a process that occurs across all tissues [1]. This indicates that mutations due to UV light follow distinct mutation patterns that are not captured by models trained on other tissues.

Another striking example were T > G substitutions in the esophagus, which were exceptionally well predictable by the esophagus-specific model (Fig. 4B, C) while other models performed worse. These T > G substitutions possibly correspond to COSMIC signature SBS17b, a mutation signature of unknown aetiology. This signature is specific to esophageal cancer and other tumors of the digestive tract [1, 21]. Even though the esophagus-specific model vastly outperformed all other models in this case, models from e.g. the lung or the skin could still better predict T > G substitutions in the esophagus than in their original tissues (e.g., note the horizontal band in Fig. 4D, T > G). This suggests that these mutations were caused by a mechanism that is strongly affected by the chromatin context or other genomic features. Supporting this notion, mutations from the SBS17b signature have a tendency to occur in hotspots [13].

Notably, extending this analysis by also considering the 3' and 5' sequence context did not change any of the conclusions above (Additional file 2: Fig. S11). In conclusion, the predictability of mutations in a tissue is mostly determined by (i) the relative frequency of specific substitutions and (ii) the predictability of each substitution type in that tissue, which in turn seems to be influenced by the mutagen. Other factors, such as the number of mutations in the training data, or the availability and quality of genomic features only minimally influence the model performance.

### Universal all-tissue model with selected predictors

**Fig. 5:**
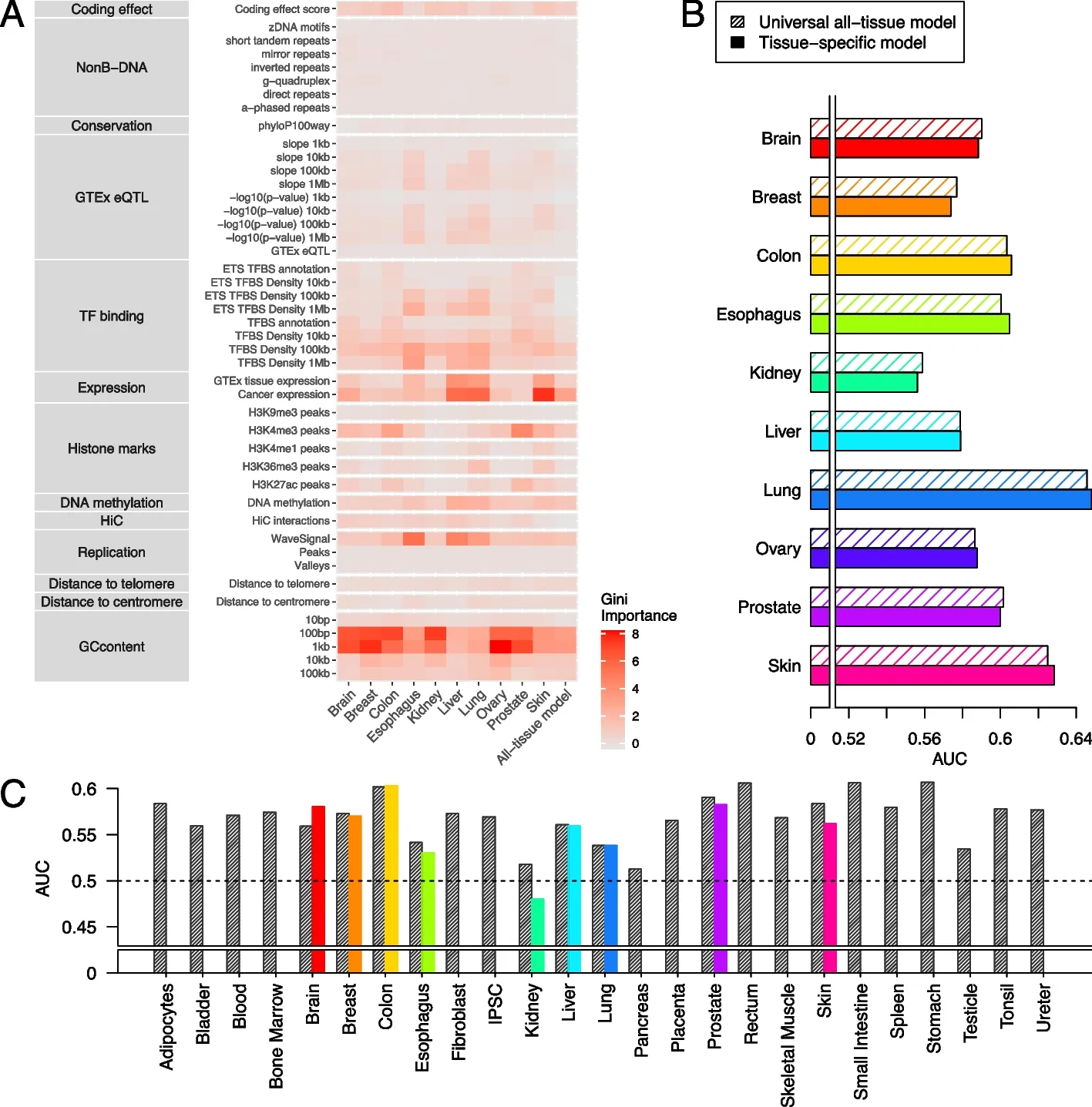
Universal all-tissue model **A** RF gini importance values of the predictors in the tissue-specific models, compared to the all-tissue universal model. **B** Prediction performance of the universal all-tissue model on each tumor tissue, compared to the respective tissue-specific models. **C** Performance (AUC) of the universal all-tissue model and the tissue-specific models on mutation data from healthy (non-tumor) tissues. The dashed grey line indicates the performance of a random classifier

Our cross-tissue analyses suggested that differences in model performance were largely driven by the underlying mutation types rather than by tissue-specific predictors. This led us to ask whether a single, universal model trained on all tissues could capture both shared and tissue-specific mutational patterns, while remaining generalizable to tissues not included in the training set. To test this, we combined mutation data and genomic feature measurements across all tissues and trained a universal ‘all-tissue model’ (Additional file 2: Fig. S12). To simplify the application of the universal all-tissue model to new tissues we restricted the feature set to the 45 predictors (out of 146) that were available in all tissues (Methods; Fig. 5A).

This universal, tissue-agnostic model performed almost as well as the tissue-specific models (AUC difference < 0.01 for each tissue; paired t-test on per-chromosome AUCs, BH-adjusted p > 0.10 for all 10 tissues; Fig. 5B), demonstrating that it effectively captured mutational patterns across tissues and that restricting the predictors did not compromise model performance. These results indicated that a single, integrated model could provide a generalizable framework for cross-tissue mutation prediction without sacrificing accuracy.

### Successful prediction of somatic mutations in healthy tissues

Next, we addressed two questions. First: could our models predict mutations in healthy (i.e. non-tumorous) tissues? And second: could the all-tissue universal model predict mutations in new tissues that were never used during the model training? We addressed both questions using 178,331 somatic mutations from 25 healthy human tissues (SomaMutDB [22], Additional file 2: Fig. S13A). The predictions were better than random for all tissues that were used for the training (mean AUC = 0.556, one-sample t-test, *p* = 1.6 × 10⁻^3^; Fig. 5C). The only exception were mutations in the kidney, where the kidney-specific model reached suboptimal performance (AUC < 0.5). This is in line with previous observations that kidney cancer mutations tend to be hard to predict [1]. However, the universal model was able to generate meaningful predictions for this tissue. Hence, even though our models were trained on mutation data from tumor specimens, they could correctly predict mutations in healthy tissues. The universal model performed comparably to the tissue-specific models across these 9 tissues (mean ΔAUC = 0.007 in favour of the universal model, paired t-test, *p* = 0.26; Fig. 5C, Additional file 2: Fig. S13B, C). Furthermore, the all-tissue generic model could predict mutations in most unseen tissues (i.e. tissues that we did not use during the training phase) better than random (one-sample t-test, *p* = 1.4 × 10⁻⁹; Fig. 5C).

The fact that the all-tissue model could predict mutations in healthy, unseen tissues demonstrates that our model learned fundamental relationships between genomic features and mutation propensities that are invariant across cell types and tissues.

### Predicting mutations outside the exome

**Fig. 6:**
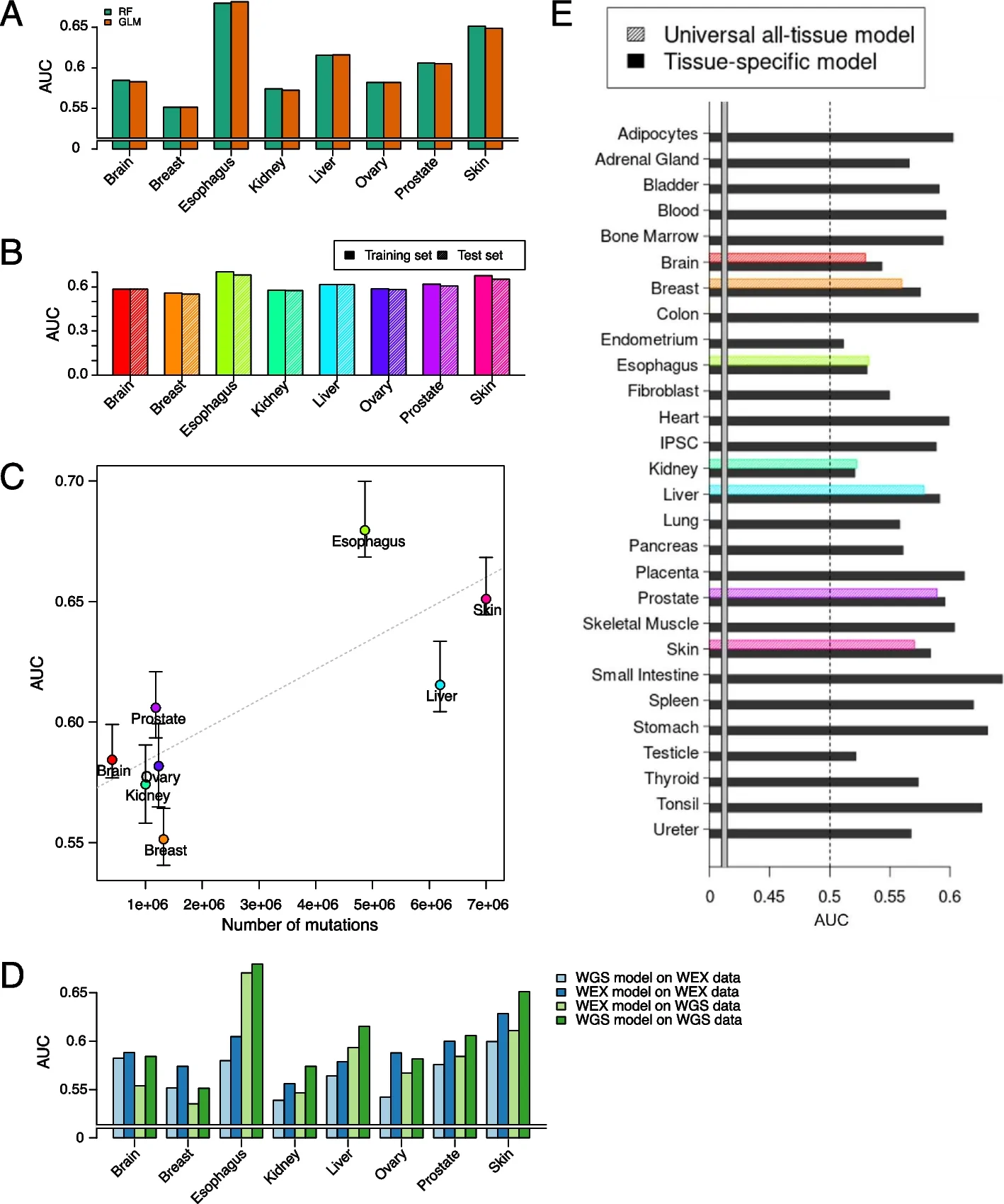
Predicting somatic mutations along the whole genome. **A** Comparison of CWCV-based performance between RF and logistic regression (GLM). **B** Comparison between test and training data performance of the RF whole genome model. **C** Dependency between model performance of each tissue and available data size. **D** We applied the whole genome model to the exome data and vice versa and computed prediction performance. For comparison, we also display AUC values achieved by each of the two models on the training data (with CWCV). **E** Performance (AUC) of the universal all-tissue model and the tissue-specific models on mutation data from healthy (non-tumor) tissues. The dashed grey line indicates the performance of a random classifier

Motivated by the success of our model for the coding exome, we generated a model for the entire human genome using mutations from the PCAWG Consortium [23]. We used the same tissues, except colon and lung, because the Pan-Cancer Atlas did not contain whole-genome mutation data for these two tissues. The data processing and model architecture remained the same. The only differences were the addition of predictors reflecting whether a position was coding/non-coding or annotated in a transcript or not, since transcriptional activity is an important feature influencing somatic mutation rates. Furthermore, since the expression value for non-transcribed genomic regions is not defined, we added predictors where expression was computed in a window around each position instead (Additional file 1: Table S17). Recapitulating earlier findings for the whole-exome models, (i) the whole-genome models predicted mutation rates better than random guessing in all tissues (one-sample t-test, BH-adjusted *p* < 1 × 10⁻^15^; Fig. 6A), (ii) the Random Forest models performed better than the linear models in four tissues (DeLong's test, two-sided, BH-adjusted *p* < 0.05 in 4 of 8 tissues; Fig. 6A), (iii) comparison of test set and training set performance confirmed the absence of overfitting (Fig. 6B), (iv) prediction performance varied greatly between tissues (Fig. 6C), and, (v) the feature importances were similar across tissues (Additional file 2: Fig. S14).

Next, we compared the performance of the whole-genome model to the exome model for predicting mutation propensities in coding parts. Even though the whole-genome model predicted mutation propensities in exonic regions better than random (mean AUC = 0.567 across 8 tissues, one-sample t-test, *p* = 4.8 × 10⁻^5^), its performance was worse than the exome-specific model for all tissues that we tested (mean ΔAUC = − 0.023, paired t-test, *p* = 8.4 × 10⁻^4^; Fig. 6D). Additionally, we also applied the exome-specific model to the whole genome (i.e. across all coding and non-coding parts), which—as expected—resulted in predictions that were worse than the whole-genome model (mean ΔAUC = − 0.023, paired t-test, *p* = 3.4 × 10⁻^4^; Fig. 6D). Thus, these analyses demonstrated that the whole-genome model successfully predicted mutation propensities outside coding regions and that distinguishing exonic and non-exonic parts of the genome during the training improved model predictions. Finally, as for the exome model, we prepared a whole-genome universal all-tissue model and tested it on a separate dataset of 28 healthy tissues. We found that the predictions were better than random (AUC > 0.5; mean AUC = 0.582, one-sample t-test, *p* = 1.4 × 10⁻^12^; Fig. 6E), both for the tissues included in training (cancer) data as well as for previously unseen tissues. For the seven healthy tissues with both universal and tissue-specific predictions, the universal model performed only marginally better than the tissue-specific models (mean ΔAUC = + 0.009, paired t-test, *p* = 0.021; Fig. 6E), demonstrating that it effectively captured mutational patterns across tissues.

### Accounting for the sequence context improves predictions at small genomic scales

**Fig. 7:**
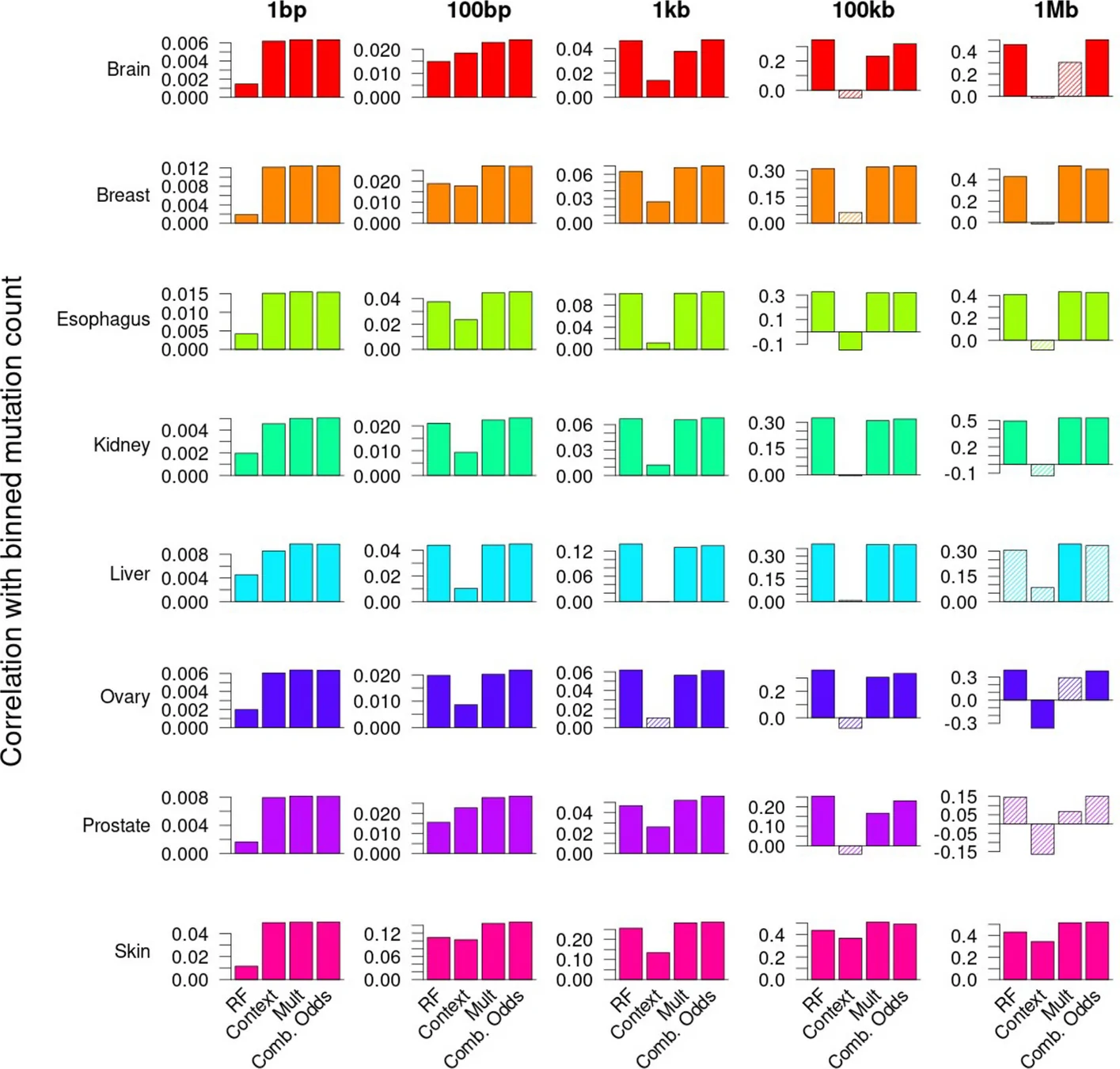
Integrating DNA sequence context with other genomic features to enhance the prediction of mutation rates. Evaluation of RF-based and sequence context-based predictions on WGS healthy tissue data in various bin sizes. Predictions of the RF-based (*RF*), sequence context-based (*Context*), and two combination approaches (*Mult*, *Comb. Odds*; see Methods for details) were averaged over bins of varying sizes and correlated (Spearman rank correlation) with observed mutation frequencies in the same bins. Bars filled with solid color indicate significant correlation with observed mutation rates (*p* < = 0.05), while striped bars represent insignificant correlation (p > 0.05)

Sequence context majorly influences mutation susceptibility along the genome and specific mutagenic processes result in mutations in specific sequence contexts. In fact, ‘mutational signatures’ (i.e. the frequency of substitution types in a specific sequence context) can be used to infer mutagens that have acted on a given tissue [1]. Our RF model was designed to be independent of sequence context by balancing mutated and matching non-mutated positions in the training data with respect to their pentamer sequence context; e.g. a mutated position with the sequence context “AC[T > A]CC” was balanced by a non-mutated position with the sequence context “ACTCC”. This strategy minimized confounding of genomic features with sequence context. Thereby it became possible to separate the effect of DNA sequence context on the mutational susceptibility from the influence of other genomic features, such as chromatin structure. To explicitly investigate the relative impact of sequence context on mutation rate variation along the genome compared to larger-scale genomic features, we first created a model that predicted mutation rates based on sequence context alone (Methods). In short, the probability of a mutation at each position was estimated as the rate of mutation for the respective sequence pentamer in the training data (i.e., the number of mutations observed at a specific pentamer in a tissue divided by the total number of occurrences of this pentamer in the genome). Next, we compared the predictive power (using again CWCV) of four modelling strategies: first, only using the RF model from above that was exclusively based on genomic features other than the sequence context (‘RF’). Second, only using the pentamer-based mutation rates, i.e. only using sequence context and no other information (‘Context’). Third, combining RF and Context by multiplying their prediction scores (‘Mult’). Fourth, combining odds ratios resulting from the two models (‘Comb Odds’, see Methods for details). To compare these four approaches we quantified the model performance by binning the human genome in bins of sizes ranging from 1 base pair to 1 Mb, computed the average mutation rate of each modelling approach across the bins and correlated those predicted rates with the observed number of mutations per bin in the test data (Fig. 7). These correlations were mostly significantly greater than zero, indicating that both the RF-based and the context-based models as well as their combinations were actually predictive across tissues and genomic bin sizes. Absolute correlation values increased with larger bin sizes for the RF model, while the sequence context-based model outperformed the RF at the single basepair resolution. This indicates that, while the sequence context, depending on the mutagens acting on the tissue, influences the local mutation propensity, large-scale mutational patterns are determined by other genomic features. Finally, the two combined approaches (‘Mult’ and ‘Comb Odds’) performed very well across all bin sizes and often even outperformed both of the single models. Similar results were obtained for the coding exome and using healthy tissues (Additional file 2: Figs. S15, 16).

The implications of these findings are as follows: when comparing two nearby nucleotides (e.g., positions separated by less than 10 bp) the sequence context alone is sufficient to determine which site is more likely to undergo mutation in a given tissue. Incorporating chromatin context provides no additional predictive power, since such closely spaced nucleotides experience essentially the same chromatin environment. In contrast, when predicting variation in mutation rates across broader genomic regions (i.e., asking which of two larger intervals is more prone to accumulating mutations), it becomes essential to account for chromatin structure and other genomic features.

## Discussion

Our integrated modelling approach quantifies the relative contribution of genomic features to mutational biases across scales and tissues. The resulting models faithfully predict mutation susceptibilities in coding and non-coding parts of the human genome, and also in tissues that were unavailable during the training phase. Models could be exchanged between tissues with only a minor loss in predictive power and they were readily applicable to healthy (non-cancerous) tissues. Further, varying the modelling strategy, such as switching between linear regression and Random Forest did not majorly affect the predictive power of the models. (We also tested XGBoost—results not shown—which yielded similar results as Random Forest.) These results suggest that fundamental, tissue-invariant relationships between the chromatin and biochemical processes dictate positional biases of mutations.

Although AUCs measuring model performance to distinguish mutated and unmutated sites were mostly significantly better than random, they were modest in size (mostly around 0.6). This was expected, because our goal was to predict mutational biases rather than individual mutations. The occurrence of mutations is a random process and it is practically impossible to predict the genomic position of the next mutation. Consequently, mutations arising outside predicted high-susceptibility regions would appear as ‘false negatives’ and depress the absolute values of ROCs and precision-recall curves. Prior studies have established that even at kilobase resolution, comprehensive genomic features explain only 70–77% of mutation rate variance [24]. At megabase resolution, 55–86% of variance can be explained [10, 11]. Single-nucleotide predictions might face the additional challenge that mutagenesis retains an inherent stochastic component arising from random polymerase errors during DNA replication [25]. Thus, the gap between our AUC and perfect discrimination (1.0) does not reflect model inadequacy but rather intrinsic stochasticity in mutagenesis. Our models faithfully captured mutational biases, identifying regions with elevated susceptibility. Importantly, those predictions were robust, since models and even model predictions could be transferred between tissues, including independent datasets and non-carcinogenic tissues not used in training. Thus, despite the seemingly low AUCs, the models performed remarkably robustly, indicating that they learn shared determinants of mutation risk– such as chromatin accessibility, replication timing, DNA damage and repair that operate similarly across cell types – rather than tissue or cancer specific confounders.

A potential concern is that predicted mutation susceptibility reflects post-hoc effects of clonal expansion or selection rather than intrinsic mutational processes. We mitigated this by excluding recurrently mutated positions from training data as they could most likely be driven by positive selection; and, by demonstrating transferability of models trained on cancer to healthy tissues, where selection is minimal. While single-cell or duplex sequencing, would more directly validate per-cell mutational processes unconfounded by selection, current single-cell somatic mutation data remains extremely sparse (~ 100 to ~ 5,000 SNVs per cell) [26] — making per-position susceptibility validation at the single-cell level statistically underpowered.

Our analysis revealed substitution types that were better predictable by our models than others (e.g., C-to-A versus C-to-G in Fig. 4D), resulting in greater model performance for tissues harboring more of these “easy-to-predict” mutations. Further, several of these substitution types were predictable by models trained on different tissues (e.g., C-to-A in Fig. 4D), hence they likely resulted from processes that are invariant across tissues. At the same time, some mutagens can cause mutations that are only predictable by tissue-specific models (e.g., a type of C-to-T mutations specific to the skin, most likely caused by UV-induced DNA damage), suggesting that these mutational mechanisms have their own ‘private’ dependencies on chromatin-related features. These findings underline the importance of the mutagen for explaining mutational biases: mutagens can be highly tissue-specific (e.g., tobacco in the lung, UV in the skin) and selectively cause mutations that are more strongly affected by the chromatin context than others, which in turn explains variable model performance across the tissues. Thus, the relative predictability of mutations in a tissue is only minorly affected by cell type-specific chromatin context and much more dependent on the mutagens.

Whereas previous work had already shown that genomic features such as sequence context and chromatin structure affect mutation rates at different scales [19], it remained elusive how much each feature contributes at which scale. Our work enables a quantitative assessment of how determinants of mutation rates impact mutation susceptibilities at different genomic scales. By analysing mutation rates and their predictability over seven orders of magnitude, we could show that local variation in mutation frequencies (on the order of dozens of base pairs) is primarily driven by the sequence context. Variation on greater scales (i.e. comparing regions of hundreds to thousands of base pairs in length) is independent of the sequence context and much more dependent on chromatin structure, gene expression and other large-scale genomic features (Fig. 7).

Thus, the integrated, multi-scale models presented here resulted in important insights into the mechanisms determining mutation frequencies and they provide a unified platform for future research addressing questions related to tumor evolution, driver detection, aging, and age-associated diseases.

## Conclusions

Our work reveals largely invariant relationships between chromatin features and mutational susceptibility across human tissues. Tissue-to-tissue variation in mutation patterns is mostly driven by the mutagen and much less so by tissue-specific gene expression and chromatin structure. Mutation rates at single-basepair resolution are best predicted by the DNA sequence context, while mutation rates at greater genomic scales are better predicted using chromatin-related features. Both aspects can be combined in joined models that are predictive across healthy human tissues, even if those were not used for model training.

## Methods

### Data collection

#### Reference genome files

All analyses were based on human genome version GrCh37 and limited to the autosomes (Additional file 1: Table S3). The coding exome, used for all exome-based analyses, was defined as follows: The annotation gtf file was filtered for exons from known protein-coding transcripts (according to feature type = "exon" and the tags type = "protein_coding", transcript_type = "protein_coding" and transcript_status = "KNOWN"). In addition, we excluded potentially problematic genomic regions such as repeats, or regions prone to mapping problems using repeatMasker, Tandem Repeats Finder, 100mer Alignability, and ENCODE [27] Consensus Excludable annotations (Additional file 1: Table S3). For the whole genome analyses, we restricted the genome to “non-problematic” regions by excluding problematic genomic intervals defined by the ENCODE Consensus Excludable Regions (Additional file 1: Table S3). Files were transformed from hg38 to hg19 using the liftOver tool from UCSC genome browser [28] where necessary.

#### Exome cancer mutations

We used mutation calls from the Pan-Cancer Atlas [29], which contains uniformly processed sequencing data from 33 tumor types profiled by TCGA (Additional file 1: Table S3). We took the subset of mutations that corresponded to SNVs (column Variant type = "SNP") and that lay in regions with sufficient coverage (column n_depth > = 8 and column t_depth > = 14). We then filtered out mutations with > 1% population frequency (in any of the ancestries GMAF, AFR_MAF, AMR_MAF, EAS_MAF, EUR_MAF, SAS_MAF, AA_MAF, or EA_MAF). Finally, we subset the variants to positions lying within non-problematic exonic regions (defined above). For each tissue, we used the variants of each corresponding cancer type (Brain: LGG, Breast: BRCA, Colon: COADREAD, Esophagus: ESCA, Kidney: KIRC, Liver: LIHC, Lung: LUAD, Ovary: OV, Prostate: PRAD, Skin: SKCM).

#### Whole genome cancer mutations

For the whole genome models, we used the mutation calls generated by the PCAWG project for ICGC cancer samples [23] (Additional file 1: Table S3). As for the exome mutations, we subsetted the mutations to SNVs (column Variant_Type = "SNP") from the autosomes. We also filtered for variants with sufficient support (at least two reads supporting the alternative allele in the cancer sample, a coverage of at least 14, and called by at least two mutation calling pipelines). We removed variants with > 1% population frequency in the 1000 genomes project (column i_1000genomes_AF). Again, we excluded mutations that were detected in more than one sample. Finally, we limited the analysis to "non-problematic" genomic regions (defined above). The remaining mutations were partitioned to each tissue (Brain: CNS-Medullo, CNS-PiloAstro, Breast: Breast-AdenoCa, Esophagus: Eso-AdenoCa, Kidney: Kidney-RCC, Liver: Liver-HCC, Ovary: Ovary-AdenoCA, Prostate: Prost-AdenoCA, Skin: Skin-Melanoma).

#### Healthy tissue somatic mutations

Healthy tissue mutations were taken from SomaMutDB V1.4 [22] (Additional file 1: Table S3). We generated two separate healthy tissue datasets: an exonic dataset for the validation of the exome-based model, and another genome-wide dataset for the validation of the whole genome model. We only used WGS-based samples for the validation of the WGS model (excluding files marked with “WES” or “exonic”). For the validation of the WES model, we used WGS as well as WES samples, but filtered mutations to exonic regions. In some studies, both WGS and WES were available for the same samples, so we used each file for the corresponding validation set only. Files from two studies were used for the WES set only [30, 31].

One study [32] contained WGS, WES and WES with high depth (hiWES), indicated by a corresponding tag in the file ID. We used only the WGS files for the whole genome dataset and used hiWES over WES files for the exome dataset, when available. For single-cell studies, where mutation calling was re-computed using the SCcaller pipeline by SomaMutDB, we used these calls instead of the original mutation calls. We removed embryonal samples and samples marked as duplicates ("T08_61F_b01_lo0048", "T08_61F_b01_lo0035", "T08_61F_b01_lo0071", "T08_61F_b01_lo0091"). Finally, we disregarded tissues that had fewer than 100 total mutations (Adrenal_gland, Endometrium, Heart and Thyroid in the exome dataset).

#### Balancing of training data mutations

The following processing steps were done for the cancer mutations and healthy tissue mutations, for the exome and whole genome, respectively. We reduced the impact of positive selection on the training data by removing positions mutated in more than one patient (Additional file 2: Fig. S1). To prepare the training data, we created a dataset with an equal number of true positive (TP) and true negative (TN) positions, which corresponded to mutated and non-mutated positions, respectively. For each observed mutation (TP), a matching TN position was randomly selected from the non-mutated part of the genome. For the exome applications, we sampled TN positions only from exonic regions (as defined above), while for the whole genome data, we sampled from all “non-problematic” genomic positions (defined above). All observed mutations (i.e., TPs before filtering) were excluded when sampling for TN positions. To limit the confounding effect of the sequence context with other genomic features, TP and TN positions were balanced with respect to sequence context pentamers (i.e., the two 5' and 3' nucleotides adjacent to each position, e.g., a mutated position with the sequence context “AC[T > A]CC” was balanced by a non-mutated position with the sequence context “ACTCC”). As a result, the proportion of each of the 512 possible pentamers (when merging reverse complement pentamers) was equivalent between the set of TPs and TNs. By creating a balanced TP/TN training data set we avoided potential issues with imbalanced training data [33].

#### Processing of predictors

In order to predict somatic mutation frequency along the genome, we collected 146 genomic features that were previously shown to be correlated with somatic mutation rate, or which had a potential mechanistic connection with somatic mutation (Additional file 1: Table S1). When possible, the predictors were tissue-specific, so that the potential tissue-specific influence of a genomic feature on the mutation susceptibility could be accounted for by the model. Further, some features were averaged (‘smoothed’) using genomic windows of varying sizes (1 bp, 10 bp, 100 bp, 1 kb, 10 kb, 100 kb, and 1 Mbp) to account for noise in the features and a possible influence of the feature on mutation susceptibility acting at a larger scale. Since a single predictor can span multiple genomic ranges, each range is treated as a separate predictor, meaning that some predictors may be correlated rather than fully independent. (Additional file 2: Fig. S17 A-J).

In order to streamline data preparation, predictors were pre-processed so that a single file encompasses the genome-wide signal of a tissue for each predictor, either in the form of a BigWig file or a GR object [34]. For instance, replicates of histone modification ChipSeq measurements for a specific tissue were processed and combined into a single BigWig file. This BigWig file was then read out to extract the signal of this histone modification for specific positions. Exceptions were the features “GC content” and “Coding effect score”, which were computed in-place for each specific position. The sources and pre-processing steps for each predictor are described below.

#### Coding effect score

This feature represents the effect a mutation at a specific genomic position would likely have on the encoded transcript or protein. To that end, we used SIFT [35]. The effect of a variant (i.e., whether it changes the protein sequence of the encoded gene or even leads to a stop-gain or stop-loss) depends on the exact type of mutation. For example, an A > T variant might be silent, while A > G leads to an amino acid exchange. Since the type of mutation was undefined for TNs, we considered every possible mutation for each query position. Therefore, for each query position, we generated three VCF entries, corresponding to the three possible nucleotide changes that could occur at this position. The resulting VCF file was used as input for SIFT. The column "VarType" of the SIFT output was then translated into a score: "NONCODING" mutations were assigned a score of 0, "SYNONYMOUS" mutations got a score of 1, "NONSYNONYMOUS" got a score of 2, and the remaining ("START-LOST", "STOP-GAIN", or "STOP-LOSS") were assigned a score of 3. The scores resulting from the three possible nucleotide changes for each position were then averaged and used as the effect score. Specific versions of all tools used are summarized in Additional file 1: Table S20.

#### Non-B DNA

Non-B DNA describes DNA stretches that differ from the canonical B-DNA conformation. Annotations of regions in hg19 that were predicted to form non-B structures were downloaded from non-B DB (Additional file 1: Table S3) [36]. The database includes positions of sequence motifs that tend to form non-B conformations, including a-phased repeats, direct repeats, g-quadruplex forming repeats, inverted repeats, mirror repeats, short tandem repeats, and Z-DNA motifs. For each of these features, we combined the annotations across all chromosomes into a GR object.

#### Conservation

A bigWig with conservation scores computed by UCSC based on multiple alignments of 99 vertebrate genomes to the human genome was downloaded from UCSC [28] (Additional file 1: Table S3) and used as-is.

#### GTEx eQTL

eQTL computed by GTEx v8 were downloaded from GTEx [37] (Additional file 1: Table S3). For each tissue, the significant variant gene pairs were converted into two GR objects, taking the eQTL locations as genomic positions and either -log(nominal *p*-value) or the eQTL slope as the value column. Since v8 GTEx is based on hg38, positions were lifted over to hg19. For the brain predictors, all GTEx brain regions were merged together into one file for *p*-values and slope, respectively.

#### Transcription factor binding site annotations

TFs are proteins that bind to DNA and regulate gene expression. We used the UCSC TFBS clusters track from ENCODE (Additional file 1: Table S3) and converted it into a GR object, with the number of cells in which each TFBS was identified as a meta column. Since ETS-type TFs were previously reported to be especially prone to mutation, we created a separate GR object with only the binding sites of TFs belonging to this family. The following TFs were considered to belong to this group: E1AF, EHF, ELF, ELF1, ELF2, ELF3, ELF4, ELF5, ELG, ELK1, ELK3, ELK4, ER71, ER81, ERF, ERG, ERM, ESE, ESE1, ESE2, ESE3, ESX, ETS, ETS1, ETS2, ETV1, ETV2, ETV3, ETV4, ETV5, ETV6, ETV7, FEV, FLI1, GABP, GABPA, MEF, NERF, NET, PDEF, PE-2, PE1, PEA3, PSE, PU.1, SAP-1, SAP1, SAP2, SPDEF, SPI, SPI1, SPIB, SPIC, TCF, TEL, and TEL2 [38].

#### Expression

We created three separate tissue-specific predictors for RNA expression: expression in cancer, expression in normal tissue from cancer studies ("Normal expression"), and expression from healthy organs from GTEx.

The cancer expression data, based on RNA-seq provided by the PanCanAtlas initiative [20], was downloaded from cBioPortal [39] for each tissue (Breast: BRCA, Colorectum: COADREAD, Esophagus: ESCA, Brain: GBM and LGG, Kidney: KIRC, Liver: LIHC, Lung: LUAD, Ovary: OV, Prostate: PRAD, Skin: SKCM; Additional file 1: Table S3). Since these files use Entrez Gene IDs, the corresponding positions were taken from the ENSEMBL database for GrCh37 provided by biomaRt [40]. For each tissue, we extracted the expression values across samples and calculated the log(median expression + 1). These were then saved as a GR object using gene coordinates from biomaRt. Note that annotation coordinates may overlap, which was accordingly handled downstream (i.e., if a query position overlapped with two gene annotations, their expression was averaged). The "normal expression", based on adjacent, healthy tissues from cancer studies; Additional file 1: Table S3), was processed in the same way as the cancer expression.

For calculating expression in healthy tissues, we downloaded median transcripts per million (TPM) per tissue from GTEx along with the gene annotation used in their pipeline, in order to get accurate gene coordinates for the expression values [37]. These gene coordinates were used to create a GR object for each tissue with the gene positions as ranges and log(median expression + 1) as value metacolumn. For the brain, all GTEx tissues from the brain were taken together and averaged, since brain location information from GTEx was much more specific than for TCGA cancer samples. Again, coordinates may overlap, which was appropriately handled downstream.

#### ChiP-seq and DNase-seq predictors from ENCODE

Suitable files were selected using the "Experiment search" feature on the ENCODE website [27], filtering for unperturbed, human samples and selecting appropriate tissues. This was done for histone modifications (assay_title = Histone ChIP-seq and assay_title = Mint-ChIP, Additional file 1: Tables S4 and S5), TF binding (assay_title = TF ChIP-seq, (Additional file 1: Tables S6 and S7), as well as DNA accessibility data based on DNAse-seq: assay_title = DNase-seq, Additional file 1: Tables S8 and S9) and ATAC-seq (assay_title = ATAC-seq, Additional file 1: Tables S10 and S11). Available histone modifications were: H3K27ac, H3K36me3, H3K9me3, H3K27me3, H3K4me3, H3K4me1, and H3K9ac; and available transcriptional regulators (TFs) were: CTCF, POLR2A, POLR2AphosphoS5, and EP300.

We downloaded the corresponding metafiles, further filtered them to exclude experiments with the keywords "extreme" or "severe" in columns Audit.ERROR or Audit.NOT_COMPLIANT. We only used files that were tagged as "released" (column File.Status) and that were either bigWig or bed files.

Bigwig files, corresponding to the signal *p*-value along the genome, and bed files, corresponding to Irreproducible Discovery Rate (IDR) thresholded peaks, were handled separately. For the bigWig files, we first grouped the experiments based on their respective ChiP-seq targets. Multiple files corresponding to the same target and tissue were combined by using wiggletools to compute the mean *p*-value across the multiple bigWig files along the genome. The resulting bedGraph files, one for each ChiP-seq target or DNA accessibility type (DNAse-seq or ATAC-seq), were then lifted over from hg38 to hg19. After sorting and merging overlapping genomic intervals (using bedtools merge), the files were then converted back to a bigWig file using bedGraphToBigWig [41].

The bed files containing the ChiP-seq or DNA accessibility peak calls were combined as follows: they were first converted into bedGraph files, sorted and overlapping intervals were merged using bedtools merge. We then combined all files corresponding to the same target and tissue by counting how many of these files indicated a ChiP-seq peak at each position (i.e., the peak "prevalence" across tissues), using bedtools unionbedg. The combined peaks were stored as a GR object, with the peak prevalence as a metadata column. These were then lifted over from hg38 to hg19 and sorted again.

To summarize, for each tissue, and each ChiPseq target or DNA accessibility type, we generated a bigWig file containing the averaged signal *p*-value, and a GR file containing the combined peak calls of all available experiments. Specific versions of all tools used are summarized in Additional file 1: Table S20.

#### DNA methylation

DNA methylation data based on deep whole-genome bisulfite sequencing was downloaded from a human methylome dataset based on deep whole-genome bisulfite sequencing [42]. We downloaded the bigWig files for each tissue (Additional file 1: Table S12). A custom script was used to average the methylation proportions at the same positions over multiple experiments in the same tissue and stored them as a GR file for each tissue.

#### Hi-C data

Chromatin conformation data based on Hi-C [43] was taken from ENCODE [27]. For each tissue, we selected suitable organ/tissue terms using the Experiment search feature on the ENCODE website (Additional file 1: Table S13). We filtered the metatable further according to column File.Status = "released". We used Hi-C data in the form of genome compartment annotations (first principle component of a principal component analysis of chromosome-wide Hi-C measurements in bigWig format) as well as mapping quality thresholded contact matrix (pair-wise chromatin contacts between genomic regions in hic file format) (Additional file 1: Table S14).

For the genome compartments, we imported each bigWig file as a GR object using the rtracklayer [34] package such that the normalized compartment score (i.e., the first principal component) was stored as a GR metadata column. The GR objects were lifted over from hg38 to hg19 and sorted. Multiple GR objects originating from the same tissue were combined into one GRL object where each list item corresponded to one Hi-C experiment. Downstream, values were averaged over experiments for each position.

#### Replication timing

Replication timing was based on Repli-seq data from ENCODE [27], where replication timing was determined by sequencing newly replicated DNA from fractions of cells in specific cell cycle phases (G1/G1b, S1, S2, S3, S4, G2). Replication patterns can be visualized as a continuous function along the genome, based on the smoothed signal of the fraction profile ("Wave signal"). Peaks and valleys (local maxima and minima, respectively) in this curve represent replication initiation and termination zones, respectively. We downloaded all files with the tags "WaveSignal", "Peaks", or "Valleys" from UCSC [28] (Additional file 1: Table S15). The "Peaks" and "Valleys" annotations were bed files, which were converted into a GR object for each cell line. The "WaveSignal" files were bigWig files which were converted into a GR object with the WaveSignal as a metadata column. We assigned each tissue the cell line that most closely matched the corresponding tissue, based on cell type and origin tissue of the cell lines as follows: SK-N-SH for brain, MCF-7 for breast, HepG2 for Liver, IMR90 for lung, and NHEK for the remaining tissues (i.e., colon, esophagus, kidney, ovary, prostate, and skin).

#### Distance to centromeres and telomeres

We downloaded genome assembly gaps and contigs for hg19 from UCSC (Additional file 1: Table S3). There is a known error for the hg19 version of this file, so that telomeres are missing for chromosome 17. Those were added manually according to the positions of flanking non-telomere regions. Telomere and centromere positions were extracted (eighth column = "telomere" or "centromere", respectively), read into R and saved as GR objects.

#### GC content

We computed GC content by extracting the genomic sequence of a window around each variant from the reference genome fasta file using bedtools [41] slop and computing GC proportion nucleotides within this window using bedtools nuc (column "X6_pct_gc" in the resulting output).

### Model training and evaluation

#### Mapping predictors to positions

First, the predictors were mapped to query genomic positions (true positives and true negatives) for each tissue using pre-processed genome-wide files (GR/GRL objects or bigWig), with GC content and effect scores computed in-place. For each predictor, values at query positions were extracted using a custom R function that takes as input the query positions and a configuration table specifying predictor files, mapping parameters, and transformation options (e.g., window size, summary measure, or distributional transformation; Additional file 1: Tables S16: whole exome and S17: whole genome). Depending on the predictor, summary measures included distance to the nearest feature, binary overlap, mean value within a window, or feature counts. The resulting dataset is a table with rows representing query positions and columns representing predictors at specified resolutions, which was used for model training and evaluation.

Next, model training and prediction were performed based on a CWCV (Chromosome-wise cross validation) scheme: for each of the 22 autosomes, we trained models on all except this chromosome and then used these models to get predictions (and estimate performance) for the excluded chromosome. In addition, we created a "final" model including all chromosomes, which was often used for downstream analyses.

#### Random forest

In this study, we used the R package ranger for RF [44]. RF models were grown with arguments importance = 'permutation', respect.unordered.factors = 'partition', probability = TRUE, scale.permutation.importance = TRUE, and defaults for all other parameters. In addition, we grew RF models with the argument importance = 'impurity_corrected'. The 'impurity_corrected' importance measure is less biased for differing numbers of categories and category frequencies between predictors, which is achieved by comparing the gini importance of each variable with gini importance values achieved by randomly introduced splits on the same variable in the RF [44, 45]. Due to these randomly introduced splits, the corresponding RF models cannot be used for prediction, they were only used to estimate predictor importance.

#### Logistic regression

We used the glm function in R with arguments family = binomial(link = "logit"). We computed predictor *p*-values were calculated using the summary function and then generated a second model with only the features that had a *p*-value < 0.05. This two-step approach was done for each CWCV fold as well as for the final model.

#### Lasso with stability selection

As a complementary approach, we also implemented LASSO [46] combined with SL (stability selection). The LASSO is a regularization method that introduces an ${l}_{1}$ penalty to the regression problem, encouraging sparsity in the coefficient estimates. In this study, SL was performed using the R package c060, with $p{i}_{thr}=0.6$, and a PCER of 0.1.

#### Sequence context-based prediction

We calculated the susceptibility of mutation based only on sequence context as follows: First, we counted the mutations that had occurred at each possible pentamer for each tissue (there are 512 possible pentamers when merging reverse complement pentamers). We then divided these counts by the total occurrence of the same pentamers in the entire exome (Additional file 1: Tables S18 and S19). These pentamer-wise frequencies of mutation were then used as a context-based "prediction" of mutation susceptibility. The steps above were performed with a CWCV strategy, for example the pentamer-specific mutation rates from chromosomes 2–22 were used to predict mutation probability based on sequence context for chromosome 1. This approach was done separately for the coding exome and the whole genome. In each case, we only considered pentamers and mutations lying in the genomic regions defined above for the coding exome and whole genome, respectively.

We also created combinations of the RF-based predictions and the context-based predictions in two different ways: (i) Multiplying the two scores (mult), and (ii) combining their odds (combOdds = odds/(odds + 1) with odds = RFpreds/(1-RFpreds)* contextPreds/(1-contextPreds).

#### Performance estimation

Performance estimations were always based on CWCV. We used the ROCR R package to compute ROC, AUC, and PR measures of prediction accuracy [47]. In some cases, we stratified the prediction by certain features (e.g., mutation type, patient of origin) before computing prediction performance. In such cases we faced the problem that these features were not defined for the TNs. Therefore, in those cases we randomly sampled TNs to match the sequence context distribution of the TPs. In other words, we paired each TP with a randomly selected TN with the same sequence context and put them in the same group for stratification.

#### Cross-tissue application

We applied the RF models trained in one tissue to the data of all other tissues and estimated the prediction performance. To that end, we used the CWCV fold which left out chromosome 1 during training, and predicted only on chromosome 1 of the foreign tissue. If a predictor was present in the tissue used for training, but missing in the foreign tissue, it was replaced with a dummy variable containing the mean value of the predictor in the training tissue. For the in-depth analysis of the impact of mutation data versus predictor data on model performance, we created cross-mapped data tables. To the TP and TN positions of each tissue, we mapped the predictors of each other tissue (e.g., we mapped predictors of the lung data to kidney TP and TN positions). That allowed us to flexibly interchange mutation, predictor, and model data between tissues.

#### Universal all-tissue model

The all-tissue model was trained on the concatenated datasets from all tissues. To that end, we restricted the predictor set to genomic features (n = 45) measured across all tissues. In the tissue-specific models, ChiPseq-based predictors had been computed as mean signal *p*-value in varying window sizes around each position as well as a binary predictor for ChiPseq peak annotations. The windowed predictors consistently demonstrated low Gini importance (Results), which is why, for the all-tissue model, we only kept ChiPseq peak annotations. The all-tissue model was trained on unique positions from each tissue, meaning if a position was mutated in more than one tissue, it was excluded.

#### Binning of genomic regions

For the evaluation of the methods’ performance at differing genomic scales, we split the exome/genome into continuous bins of varying size. The exome was split into 100 bp, or 1kbp bins, since larger bins were not possible because they were limited by exon sizes. For the whole genome, we used only genomic regions on chromosome 22 (for computational reasons) defined as “unproblematic” (see above), and split them into bins of sizes 100 bp, 1 kb, 100 kb, or 1 Mb. We only kept bins covering the indicated window size (i.e., bins that were not the full length, for example at the end of each exon, were filtered out). In each bin, we aggregated the prediction values of the RF-based predictions, the context-based predictions and their combinations described above by taking their mean. Accordingly, we computed the observed number of mutations (either cancer mutations or healthy tissue mutations) within each bin. We then computed the Spearman rank correlation between each binned predictor and the binned mutation counts. Correlation significance was determined using the cor.test function in R.

#### Preparing bigWig files

We converted the processed predictions for exome and genome to bigWig format for efficient visualization in genome browsers. To that end, we converted chromosome-specific data to GRanges objects using the rtracklayer package, with genomic coordinates referenced to the hg19/GRCh37 human genome assembly. Objects were exported as bigWig files, generating separate track files for each chromosome. We subsequently merged individual chromosome tracks to create genome-wide bigWig files for tissue-specific mutation susceptibility visualization. These were deposited to Zenodo with 10.5281/zenodo.21805107, and can be viewed on the UCSC genome browser using the instructions from the README file on Zenodo.

### Software and packages

All analyses were performed using publicly available software and packages in R v4.3.1 [48], including data processing, statistical modeling, and visualization. Genomic data were handled using established Bioconductor frameworks, and machine-learning models were implemented with widely used R packages. Reproducibility was ensured through version-controlled environments (conda and github). We used DeLong's test [49] for paired ROC curves (two-sided, implemented in the R package pROC [50]) to compare model performances. Multiple testing correction was performed using Benjamini & Hochberg method [51]. A detailed list of all software used with their specific versions is provided in Additional file 1: Table S20.

## Supplementary Information

Additional file 1: Tables S1-S20.

Additional file 2: Figs. S1-S17.

## Data Availability

All data used in this study were obtained from publicly available databases, such as TCGA (https://www.cancer.gov/tcga), PCAWG (https://ega-archive.org/studies/EGAS00001001692?page=0%252C2) [23], GTEx (https://gtexportal.org/home/) [37], cBioportal (https://www.cbioportal.org/) [39], and ENCODE (https://www.encodeproject.org/) [27], as cited and indicated in the methods. DNA methylation data used was from Loyfer et al., 2023 (https://doi.org/10.1038/s41586-022-05580-6) [42]. All code used for the analyses and to prepare the figures for this manuscript can be found at Zenodo https://doi.org/10.5281/zenodo.21806414 [52] and on GitHub [53]. Whole exome per tissue as well as all-tissue integrated exome and genome mutation susceptibilities can be viewed on the UCSC genome browser using bigWig files deposited at Zenodo https://doi.org/10.5281/zenodo.21805107 [54].
