# Supplementary material for: Determinants of mutation susceptibility along the genome are largely invariant across human tissues
Source: Genome Biol. 2026 Aug 22;27:268. doi: 10.1186/s13059-026-04245-1 (PMC13501610; doi:10.1186/s13059-026-04245-1)
Supplement: Supplementary file 1 [file 13059_2026_4245_MOESM2_ESM.docx]

**Fig. S1: Proportion of positions per tissue that were removed due to recurrent mutations.** We removed all mutations that were found in more than one sample, in order to limit the impact of positively selected mutations. The number of recurrent mutations is proportional with available data size. Thus, the proportion of recurrent mutations ranged between 1.13 and 22.73 percent.

**Fig. S2:** **Overview of per tissue mutation data used in this study.** Upper panel: number of mutations observed per patient for each cancer type. Horizontal lines represent the median. Lower panel: proportion of mutation types for each tissue. The six possible classes of nucleotide substitution (C>A, C>G, C>T, T>A, T>C, and T>G) were further stratified by trimer sequence context (i.e., the immediate 5' and 3' nucleotide neighbors). Merging reverse complement equivalents results in the 96 possible mutation types depicted here. Thus, each bar represents the percentage of mutations that correspond to each mutation type. Figure design adapted from Lawrence, M. S. et al., *Nature* 499, 214–218 (2013), <https://doi.org/10.1038/nature12213>.

D

**Fig. S3: Performance comparison of model approaches**. (A) AUC estimates for each of the CWCV folds (light dots) and the estimate based on the entire exome (concatenation of CWCV folds, filled dots) for the three models. (B) PR curves achieved by each model. The inset shows a magnification of the indicated left-most part of the curve in order to highlight the difference between the approaches. (C) Sina plot and overlayed boxplot of the prediction values stratified by the true TP/TN labels. The horizontal dashed line indicates a hypothetical cutoff at 0.5. (D) Test versus training performance of each model. Performance on test data was obtained via chromosome-wise cross-validation. Predictions over chromosomes were combined to compute AUC.

**Supplementary Figure 4**: Relationship between model performance (AUC) and either data size (number of TPs and TNs remaining after excluding each chromosome), total size of the coding regions of each chromosome, and the proportion of coding positions on each chromosome. Each point is one chromosome.

**Fig. S5: Comparison of chromosome-wise cross-validation predictor importance values**. Heatmap visualization of RF gini importance measures across CWCV iterations. In each of the tissue panels, each row represents one predictor and each column one of the 22 CWCV iterations. The last column in each tissue always represents the final model that includes all chromosomes. Importance measures were scaled between tissues for better comparison. Grey areas indicate predictors that were not available for a tissue. For features where peak measurements exist, their values are shown in black in the feature annotation bar. For a single feature the order is peak measurements first, followed by predictor measurements at respective genomic scales.

**Fig. S6:** Difference of RF model performance between tissues: (A) ROC and (B) Precision-recall curves achieved by each tissue.

**Fig. S7:** (A) Overview of the number of mutations available for each tissue in our data. The horizontal dashed line represents the lowest number of mutations available (i.e., kidney) which all other tissues were downsampled to. (B) Overview of the number of samples/patients making up the data for each tissue. The horizontal dashed line represents the lowest number of samples available across tissues (i.e., ovary) which was used to downsample all other tissues. (C) Prediction performance (AUC) of the models trained on either the full data for all tissues, data downsampled to match the number of mutations between tissues, or data downsampled to match the number of samples between tissues.

**Fig. S8: Effect of outlier hypermutator samples.** (A) Distribution of numbers of mutations across tissues for each tissue. The inset lists the proportion of data that the five samples with the most mutations ("hypermutators") contribute in each tissue. (B) Effect on prediction performance after removing each of the top five hypermutators from the test data. Sample 1 in brain (TCGA-DU-6392) is the only sample noticeably influencing AUC (C) Effect on model performance when removing brain sample TCGA-DU-6392 from the test data (i.e., model trained on all samples, but tested on all except TCGA-DU-6392), from the train data (i.e., model trained on all samples except TCGA-DU-6392, but tested on the full data), or both.

**Fig. S9: Mutation type distribution of hypermutators:** (A) brain

**Fig. S9: Mutation type distribution of hypermutators:** (B) breast

**Fig. S9: Mutation type distribution of hypermutators:** (C) colon

**Fig. S9: Mutation type distribution of hypermutators:** (D) esophagus

**Fig. S9: Mutation type distribution of hypermutators:** (E) ovary

**Fig. S9: Mutation type distribution of hypermutators:** (F) prostate

**Fig. S9: Mutation type distribution of hypermutators:** (G) skin

**Fig. S9: Mutation type distribution of hypermutators:** (A) brain, (B) breast, (C) colon, (D) esophagus, (E) ovary, (F) prostate, (G) skin: This figure depicts the effect of hypermutators (making up at least 5% of all mutations in this tissue) on the mutation type distribution. The mutation type distribution is defined as the frequency of the six possible classes of nucleotide substitution (C>A, C>G, C>T, T>A, T>C, and T>G) which were further stratified by trimer sequence context (i.e., the immediate 5' and 3' nucleotide neighbors). Depicted are, from left to right: the distribution of the entire tissue data, and then, for the hypermutated samples, the distribution of only the samples, the COSMIC signature that most correlated with the mutation spectrum of these samples (with Pearson's R), and the distribution of the tissue data after removing each sample, respectively.

**Fig. S10**: (A) Same as in Main Fig. 3 A (shown here for reference). Model performance (AUC based on CWCV, fill color) of RF models trained on one tissue (x-axis) to the data of all other tissues (y-axis). The numbers in the heatmap fields indicate the row-wise rank of the AUC value with lower numbers corresponding to best performance. For each tissue, we row-wise ranked the AUC values of models trained on predictors from the 10 tissues, assigning ranks from 1 to 10, with lower ranks indicating better performance. (B) Same as (A), but after downsampling test and training data to match the tissue with the fewest mutations (kidney).

**Fig. S11**: Dependence of model performance on mutation context. Each tissue-specific model was tested on the subset of positions corresponding to a certain mutation type (i.e., substitution type combined with the sequence context comprising the 3' and 5' neighbors). Each row corresponds to one such mutation type, each column to one tissue. The last two columns indicate the row-wise mean and median, respectively, for easier interpretation. White fields indicate tissue-mutation type pairs with insufficient data to compute stable AUC (i.e., fewer than 100 data points).

**Fig. S12**: Mutation spectrum of combined tissue data used to train the universal ‘all-tissue model’. Depicted are the mutation spectra of each individual tissue as well as the combination of all tissue-specific datasets. The mutational profiles count the proportions of the six substitution subtypes (C>A, C>G, C>T, T>A, T>C, and T>G) combined with the nucleotides immediately 5’ and 3’ to the mutation. Figure design adapted from (M. S. Lawrence et al., 2013).

**Fig. S13: Model performance of the exome-based universal and tissue-specific models on healthy tissues.** (A) Number of mutations aggregated across healthy tissue samples. (B) Precision-recall curves for each tissue, showing the performance of the universal all-tissue model on the healthy tissue dataset (solid black line) and on the training data (dashed black line). For tissues where corresponding cancer tissue mutation data were available during training, performance of the tissue-specific model on the healthy tissue dataset (solid colored line) and on the training data (dashed colored line) is additionally shown. (C) Same as (B), but showing ROC curves (TPR - true positive rate vs. FPR - false positive rate). The dashed grey diagonal indicates the performance of a random classifier.

**Fig. S14:** Scaled gini importance values across tissues for the Whole Genome RF model.

**Fig. S15:** Evaluation of RF-based and context-based predictions on exome data in various bin sizes. Scores of RF-based, context-based, and four combination approaches were averaged over bins of varying sizes and correlated (Spearman correlation) with cancer mutation counts observed in the same bins. All correlations were significant (p < 0.05).

**Fig. S16:** Evaluation of RF-based and context-based predictions on WGS healthy tissue data in various bin sizes. Scores of RF-based, context-based, and two combination approaches were averaged over bins of varying sizes and correlated (Spearman correlation) with mutation counts observed in the same bins. Bars filled with solid color indicate significant correlation (p <= 0.05 Spearman correlation) while striped bars represent non-significant correlation (p > 0.05). The sparsity of healthy tissue mutations lead to inconclusive results.

**Fig. S17: Correlation between predictor variables of training data:** (A) brain

**Fig. S17: Correlation between predictor variables of training data:** (B) breast

**Fig. S17: Correlation between predictor variables of training data:** (C) colon

**Fig. S17: Correlation between predictor variables of training data:** (D) esophagus

**Fig. S17: Correlation between predictor variables of training data:** (E) kidney

**Fig. S17: Correlation between predictor variables of training data:** (F) liver

**Fig. S17: Correlation between predictor variables of training data:** (G) lung

**Fig. S17: Correlation between predictor variables of training data:** (H) ovary

**Fig. S17: Correlation between predictor variables of training data:** (I) prostate

**Fig. S17: Correlation between predictor variables of training data:** (J) skin

**Fig. S17: Correlation between predictor variables of training data:** (A) brain, (B) breast, (C) colon, (D) esophagus, (E) kidney, (F) liver, (G) lung, (H) ovary, (I) prostate, (J) skin. Pearson correlation coefficient between each predictor pair is represented by dot size and color. For features where peak measurements exist, their values are shown in black in the feature annotation bar. For a single feature the order is peak measurements first, followed by predictor measurements at respective genomic scales.
